# Lack of Functional P110δ Affects Expression of Activation Marker CD80 but Does Not Influence Functions of Neutrophils

**DOI:** 10.3390/ijms23126361

**Published:** 2022-06-07

**Authors:** Aneta Manda-Handzlik, Agnieszka Mroczek, Weronika Kuźmicka, Adrianna Cieloch, Zuzanna Homoncik, Angelika Muchowicz, Urszula Demkow, Małgorzata Wachowska

**Affiliations:** 1 Department of Laboratory Diagnostics and Clinical Immunology of Developmental Age, Medical University of Warsaw, Zwirki i Wigury 63a Street, 02-091 Warsaw, Poland; aneta.manda-handzlik@wum.edu.pl (A.M.-H.); agnieszka.mroczek@wum.edu.pl (A.M.); weronika.kuzmicka@wum.edu.pl (W.K.); adrianna.cieloch@wum.edu.pl (A.C.); homoncik.zuzanna@gmail.com (Z.H.); urszula.demkow@uckwum.pl (U.D.); 2 Doctoral School, Medical University of Warsaw, Zwirki i Wigury 61 Street, 02-091 Warsaw, Poland; 3 Student’s Scientific Group at Department of Laboratory Diagnostics and Clinical Immunology of Developmental Age, Medical University of Warsaw, 02-091 Warsaw, Poland; 4 Department of Immunology, Medical University of Warsaw, Jana Nielubowicza 5 Street, 02-097 Warsaw, Poland; angelika.muchowicz@wum.edu.pl; 5 Department of Clinical Immunology, Medical University of Warsaw, Nowogrodzka 59 Street, 02-006 Warsaw, Poland

**Keywords:** degranulation, neutrophils, neutrophil extracellular traps, p110δ subunit, phagocytosis, phosphoinositide 3-kinase, reactive oxygen species

## Abstract

Neutrophils are specialized immune cells that are essential constituents of the innate immune response. They defend the organism against pathogens through various mechanisms. It was reported that phosphatidylinositols are key players in neutrophil functions, especially in the activity of class-I phosphoinositide 3-kinases (PI3Ks). P110δ, one of the PI3K subunits, is mostly expressed in immune cells, and its activity plays an important role in inflammatory responses. The aim of this study was to investigate the role of p110δ in neutrophil antimicrobial functions, activation status and cytokine production. To this end, we used bone marrow and splenic neutrophils isolated from a murine model expressing catalytically inactive p110δ^D910A/D910A^. The level of phagocytosis and degranulation, the expressions of activation markers and cytokine production were determined by flow cytometry. ROS generation and NET release were assessed by fluorometry and fluorescent microscopy. We observed a significantly higher percentage of CD80-positive cells among the splenic granulocytes and found granulocytes subpopulations of differing phenotypes between WT and p110δ^D910A/D910A^ mice by multiparametric tSNE analysis. Moreover, we detected some differences in the expressions of activation markers, intracellular production of cytokines and bacterial killing. However, we did not observe any alterations in the selected neutrophil functions in p110δ mutant mice. Altogether, our data suggest that the catalytic p110 subunit(s), other than p110δ, is a key player in most neutrophil functions in mice. A follow-up study to correlate these in vitro results with in vivo observations is highly recommended.

## 1. Introduction

Neutrophils are terminally differentiated cells of myeloid lineage, produced in bone marrow and then released into the blood circulation. These cells migrate to the site of infection upon various inflammatory signals [1]. Neutrophils are crucial components of the innate arm of immune response, defending the host against bacterial infections by means of phagocytosis, degranulation, reactive oxygen species (ROS) and neutrophil extracellular trap (NET) formation. It has become evident that phospholipid regulation by various lipid kinases, phosphatases and lipases plays an important role in many neutrophil functions [2]. Class-I phosphoinositide 3-kinases (PI3Ks) are crucial signal transducers in granulocytes. Class I PI3Ks are heterodimers consisting of regulatory (p50, p55, p85 or p101) and catalytic p110 subunits (α, β, γ, δ). They selectively phosphorylate phosphatidylinositol 4,5-bisphosphate (PI(4,5)P2) to form phosphatidylinositol 3,4,5-trisphosphate (PI(3,4,5)P3, usually referred to as ‘PIP3′). Class I PI3Ks are regulated directly or indirectly by cell surface receptors and play important roles in early signal transduction events [3]. P110δ was the last to be described among the p110 subunits and was characterized by Chantry et al. in 1997. P110δ reveals various common features with α and β subunits, but it has divergent biochemical and structural properties and a tissue distribution mostly limited to leukocytes [4].

Although class I PI3Ks have been implicated in neutrophil inflammatory responses, there are limited data concerning the involvement of individual subunits in neutrophil functions. Our interests were directed to p110δ, as this subunit is mostly expressed in immune cells and has been shown to be important for neutrophil activation [1]. In this study, we thoroughly investigated the role of p110δ in neutrophil functions and activation. To this end, we employed a murine model expressing a catalytically inactive form of p110δ (p110δ^D910A/D910A^) mutant mice. Hence, we are the first to comprehensively analyse neutrophil functions in p110δ mutant mice; we studied phagocytosis, degranulation, ROS production and NET formation, as well as the neutrophil ability to kill bacteria. Moreover, we analysed the p110δ^D910A/D910A^ neutrophil activation status and cytokine production and compared granulocyte phenotypes between p110δ^D910A/D910A^ and WT mice by tSNE analysis.

## 2. Results

### 2.1. P110δ^D910A/D910A^ Mice Presented Unaltered White Blood Cell Percentages in Peripheral Blood

**Figure 1:**
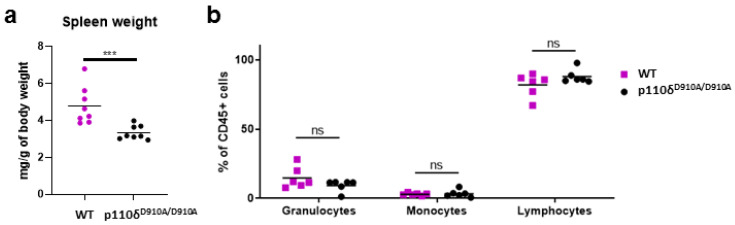
(**a**) Reduced weight of spleens in p110δ^D910A/D910A^ mice. The graph shows weight of spleens in p110δ^D910A/D910A^ mice (black dots) compared with their respective wild-type littermates (WT, purple dots). The spleen weights are expressed as mg/g of body weight. Results are shown as means with individual values. Mann–Whitney analysis disclosed significant differences between groups; n = 8. (**b**) Flow cytometry analysis of white blood cell percentages in the blood of p110δ mice (black dots) compared with their respective wild-type littermates (purple dots). The values are expressed as percentage of CD45+ cells. Statistical significance was determined by two-way ANOVA followed by Sidak multiple comparison test; n = 6. Results are shown as means with individual values. Ns—non-significant ( *p* > 0.05), *** *p* ≤ 0.001.

In accordance with a previous report [5], we observed that spleens from p110δ^D910A/D910A^ mice were significantly smaller than those of their control littermates (Figure 1a and Figure S1a) (*p* ≤ 0.001). In order to evaluate whether changes in cell accumulation in the spleens of p110δ^D910A/D910A^ mice were accompanied by altered percentages of peripheral blood leukocytes, especially myeloid cells, we examined the CD45+ population and divided it into three groups based on the expressions of the following surface antigens: CD11b, Ly6G and Ly6C (Supplementary Figure S1b). Accordingly, populations of granulocytes, monocytes and lymphocytes could be clearly distinguished. Nevertheless, no differences in the percentage of granulocytes, monocytes nor lymphocytes among peripheral blood leukocytes could be observed (*p* > 0.05) (Figure 1b) in p110δ^D910A/D910A^ mice in comparison with WT mice.

### 2.2. Neutrophils of P110δ^D910A/D910A^ Mice Were Characterized by Unaltered Granule Mobilization, Phagocytic Ability, ROS Synthesis and NET Formation

To elucidate the role of the p110δ subunit of class I PI3K in murine neutrophils, we isolated granulocytes from the bone marrow of both KO and WT mice and analysed a range of antimicrobial functions (degranulation, phagocytosis, ROS synthesis and NET formation) in these cells.

**Figure 2:**
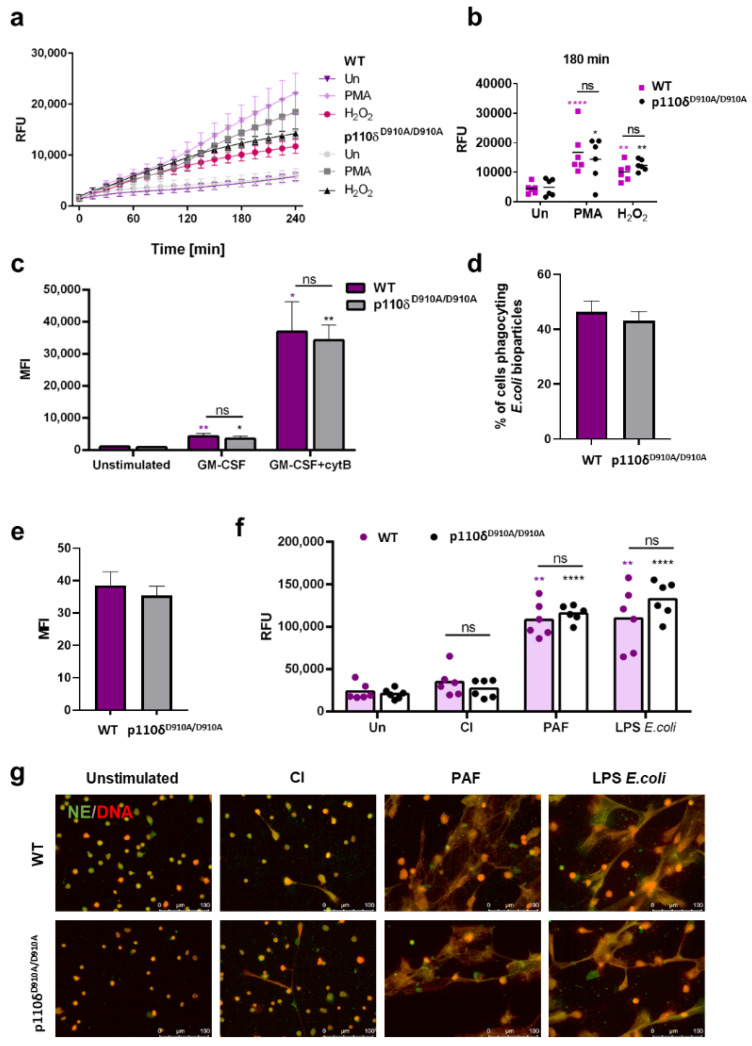
Neutrophils isolated from p110δ^D910A/D910A^ mice exhibited unaltered antimicrobial functions. (**a**,**b)** To determine ROS production, bone marrow neutrophils were loaded with dihydrorhodamine 123, stimulated with phorbol 12-myristate 13-acetate (PMA) or H_2_O_2_, and fluorescence was monitored every 15 min for 4 h (**a**); ROS production at the time point of 180 min is presented for each mouse in each group (**b**). (**c**) To assess degranulation, neutrophils were primed with granulocyte-macrophage colony-stimulating factor (GM-CSF) for 15 min, incubated with cytochalasin B (cytB) for the following 5 min and then stimulated with complement component 5a (C5a) for 15 min. After labelling with anti-Ly-6G and anti-CD63, cells were analysed by flow cytometry. (**d**,**e**) Neutrophils were incubated with *E. coli* bioparticles for 30 min, and phagocytic abilities were analysed using flow cytometry as a percentage of FITC-positive cells (**d**) and as fluorescence intensity of phagocyting cells (**e**). (**f**,**g**) NET release after 3 h of stimulation with 2 µM calcium ionophore A23187 (CI), 5 µM platelet activating factor (PAF) or 20 µg/dL lipopolysaccharides (LPS) isolated from *E. coli* was assessed fluorometrically (**f**) and microscopically after immunolabeling (**g**); one representative out of two independent experiments is presented (**g**). (**a**–**c**) Results are shown as means + SEM (**a**,**c**) or means with individual values (**b**) and were analysed by one-way ANOVA (**c**) or two-way ANOVA (**a**,**b**) with appropriate post hoc tests: Dunnett’s test for comparisons within p110δ^D910A/D910A^ or WT group and Sidak test for comparisons between p110δ^D910A/D910A^ and WT groups; n = 6. (**d**,**e**) Means + SEM are shown; data were analysed by Mann–Whitney test; n = 3. (**f**) Results are presented as means with individual values and were analysed by one-way ANOVA with post hoc Holm–Sidak test (comparisons within p110δ^D910A/D910A^ or WT group) or Friedman test with post hoc Dunn’s test (comparisons between p110δ^D910A/D910A^ and WT groups); n = 6. * *p* ≤ 0.05, ** *p* ≤ 0.01, **** *p* ≤ 0.0001. (**b**,**c**,**f**) Violet asterisks—vs. unstimulated WT mice; black asterisks—vs. unstimulated p110δ^D910A/D910A^ mice. WT—wild type mice; p110δ^D910A/D910A^—mutant mice; Un—unstimulated; MFI—mean fluorescence intensity; RFU—relative fluorescence units; ns—non-significant.

First, we evaluated the capacity of p110δ^D910A/D910A^ mouse neutrophils to form ROS by fluorometry using dihydrorhodamine 123, a dye that detects superoxide ions following oxidation to its fluorescent derivative rhodamine 123. We used PMA and H_2_O_2_ to stimulate ROS formation in cells (Figure 2a). The effect of H_2_O_2_ was statistically significant (*p* ≤ 0.01) and comparable in both study groups. Similarly, PMA strongly induced ROS release by neutrophils isolated from either type of mice (*p* ≤ 0.0001 in WT and *p* ≤ 0.05 in KO). Interestingly, the results of PMA-induced ROS production are highly heterogenous in both studied groups (Figure 2b).

Next, we studied the degranulation of neutrophil azurophilic granules by analysing an increase in the expression of surrogate marker CD63 on the plasma membrane. After priming cells with granulocyte macrophage-colony stimulating factor (GM-CSF), cytochalasin B was added to enhance the depolymerization of actin. The flow cytometry analysis did not reveal any alteration in the degranulation of neutrophils isolated from p110δ^D910A/D910A^ mice as compared with neutrophils isolated from WT mice (*p* > 0.05) (Figure 2c).

In order to study if p110δ^D910A/D910A^ mouse neutrophils differed in their phagocytic abilities, we applied flow cytometry to analyse the percentage of neutrophils phagocyting *E. coli* bioparticles; furthermore, we analysed the mean fluorescence index for each cell reflecting the amount of bioparticles phagocytosed by granulocytes. We did not observe any differences in phagocytic capacities between neutrophils isolated from p110δ^D910A/D910A^ or WT mice (*p* > 0.05) (Figure 2d,e).

Next, we analysed whether the p110δ subunit plays a role in NET formation. The analysis of neutrophil extracellular trap formation by fluorescence microscopy (Figure 2g), confirmed by the fluorometric measurement of released DNA (Figure 2f), revealed that the PAF and lipopolysaccharides (LPS) isolated from *E. coli* had the same effectiveness in stimulating NET release in both studied groups (*p* ≤ 0.01 for WT and *p* ≤ 0.0001 for p110δ^D910A/D910A^ mice). Fluorometric measurements indicated that calcium ionophore A32187 (CI) failed to induce NET release in both WT and p110δ^D910A/D910A^ mice. Accordingly, only a small fraction of cells releasing NETs in CI-stimulated samples could be observed by fluorescent microscopy.

Finally, we studied the antimicrobial capabilities of neutrophils isolated from WT and p110δ^D910A/D910A^ mice. To establish the potential of granulocytes to kill bacteria extracellularly, we incubated neutrophils with *E. coli* bacteria for 1 h and then plated the collected supernatants on agar plates. Intriguingly, the granulocytes of p110δ^D910A/D910A^ mice tended to be less effective in eradicating bacteria than the granulocytes of WT mice. The survival of *E. coli* in the presence of WT neutrophils was reduced by ca. 20% on average compared with the survival of *E. coli* in the presence of KO neutrophils (Supplementary Figure S2). Notably, although this trend in data was noticeable, it did not reach statistical significance.

### 2.3. Neutrophils of P110δ^D910A/D910A^ Mice Revealed Unaltered Cell Survival In Vitro

**Figure 3:**
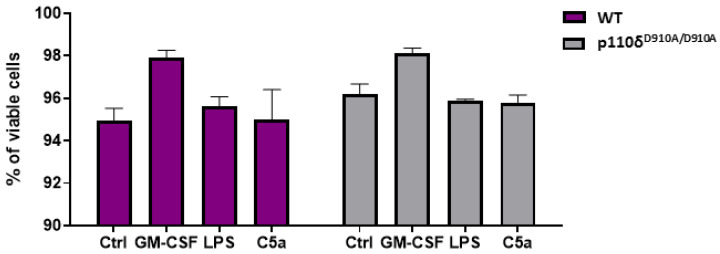
Functional knock-out of p110δ did not alter survival time of granulocytes. Isolated granulocytes were cultured in the presence of lipopolysaccharides isolated from *E. coli* (LPS), GM-CSF or C5a for 24 h; dead cells were stained by propidium iodide, and viability was assessed using flow cytometry. Means + SEM are shown; data were analysed by two-way ANOVA with post hoc Sidak test (comparisons between KO and WT groups) and one-way ANOVA with post hoc Dunnett’s test (comparisons within KO and WT groups); n = 3. WT—wild type; p110δ^D910A/D910A^—mutant mice; GM-CSF—granulocyte macrophage-colony stimulating factor; C5a—complement component 5a.

We analysed the survival of isolated granulocytes after incubation separately with LPS (isolated from *E. coli*), GM-CSF or C5a for 24 h. Following incubation, cells were stained with propidium iodide to differentiate between living and dead cells (Supplementary Figure S3). The quantification of viable cells by the flow cytometry method indicated no differences between p110δ^D910A/D910A^ and WT mice (*p* > 0.05) (Figure 3). Furthermore, among the aforementioned factors, which were previously used as pro-survival agents [6], only GM-CSF prolonged neutrophil survival both in WT and p110δ^D910A/D910A^ mice.

### 2.4. Percentage of CD80- and CD86-Positive Cells Was Lower in P110δ^D910A/D910A^ Mice than in WT Mice

**Figure 4:**
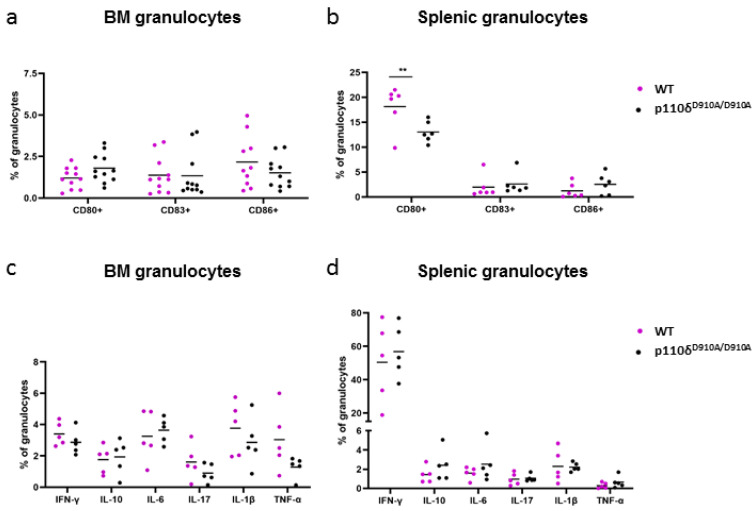
(**a**,**b**) Flow cytometry analysis of neutrophil activation markers; p110δ^D910A/D910A^ mice (black dots) were compared to their respective WT littermates (purple dots). Neutrophils isolated from bone marrow (**a**) and spleen (**b**) were analysed for their expressions of CD80, CD83 and CD86. (**c**,**d**) Flow cytometry analysis of ex vivo stimulation of cells and intracellular cytokine staining; p110δ^D910A/D910A^ mice (black dots) were compared to their respective WT littermates (purple dots). Neutrophils isolated from bone marrow (**c**) and spleen (**d**) were analysed for their intracellular levels of IFN-γ, IL-10, IL-6, IL-17, IL-1β and TNF-α. The graphs are expressed as percentage of granulocytes synthesizing each cytokine. Statistical significance was determined by two-way ANOVA followed by Sidak multiple comparisons test; (**a**) n = 11 (**b**) n = 6 (**c**) n = 5 (**d**) n = 5, ** *p* ≤ 0.01. Results are shown as means with individual values.

**Figure 5:**
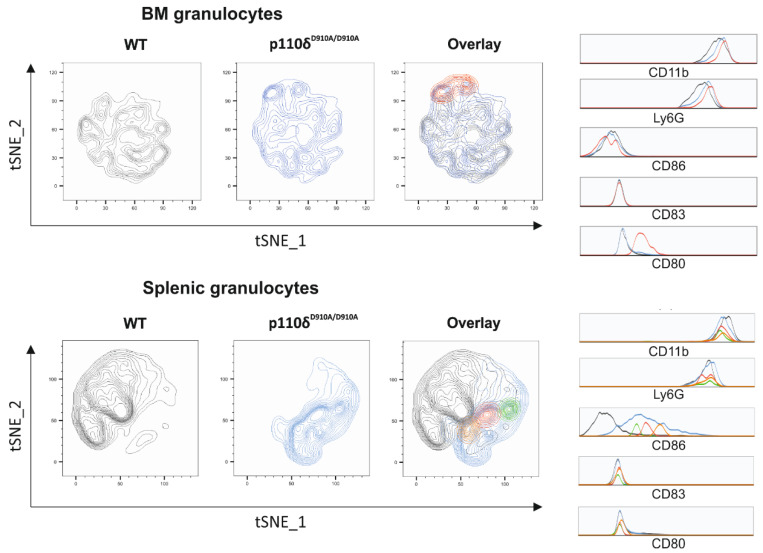
tSNE analysis of the phenotype of granulocytes isolated from WT (grey) and p110δ^D910A/D910A^ (blue) mice. (**above**) Analysis of granulocytes isolated from bone marrow (BM). The overlay of counterplots presents the BM granulocyte subpopulation, specific for p110δ^D910A/D910A^ mice (red). (**below**) Analysis of granulocytes isolated from the spleen. The overlay of counterplots presents three distinct subpopulations, specific for p110δ^D910A/D910A^ mice (red, orange and green). The granulocytes were plotted on the graphs according to the expressions of CD83, CD86 and CD80; expressions of these markers for each subpopulation is presented on the histograms. The counter plots show the results of the analysis of 3 WT and 3 p110δ^D910A/D910A^ representative mice.

To further elaborate on the role of the p110δ subunit in granulocytic functions, we analysed the expressions of activation markers and the production of cytokines by the residual bone marrow (BM) and splenic neutrophils of WT and p110δ^D910A/D910A^ mice (the gating strategy is presented in Supplementary Figure S4). The flow cytometry analysis of neutrophil activation markers revealed a significantly higher percentage of CD80-positive cells in WT than p110δ^D910A/D910A^ mice among splenic neutrophils. In addition, we observed a slight increase in the percentage of CD86-positive cells among splenic granulocytes of p110δ^D910A/D910A^ as compared with WT mice (Figure 4b). Moreover, in the BM neutrophils of p110δ^D910A/D910A^, we found a higher percentage of CD80-positive cells and a lower percentage of CD86-positive cells than in WT; however, these data did not reach statistical significance (Figure 4a). Yet, we did not observe any differences between WT and p110δ^D910A/D910A^ mice regarding the expression of CD83 on the splenic or BM neutrophil surface. To further analyse the activation status of granulocytes in WT and p110δ^D910A/D910A^ mice, we employed multiparametric tSNE (t-distributed stochastic neighbour embedding) analysis. The obtained graphs (Figure 5) present the subpopulations of granulocytes found in BM and in the spleen, specific for p110δ^D910A/D910A^ mice (blue counterplots) and WT mice (grey counterplots), distinguished based on the expressions of CD80, CD83 and CD86 markers. The tSNE analysis of BM granulocytes revealed the presence of a subpopulation with increased expression of CD80 in p110δ^D910A/D910A^ mice as compared with WT granulocytes (Figure 5a). Additionally, the tSNE analysis of splenic granulocytes identified three subpopulations with differing expression of CD86, distinct for p110δ^D910A/D910A^ mice, and the expression of CD86 in these cells was higher than in the granulocytes of WT mice (Figure 5b).

Further flow cytometry analyses indicated a slightly lower, although statistically insignificant, production of IL-17, IL-1β and TNF-α by BM granulocytes of p110δ^D910A/D910A^ mice in comparison with WT mice. Yet, we did not detect any differences in the intracellular production of cytokines, after stimulation with PMA, by splenic granulocytes between the studied groups (Figure 4c,d).

## 3. Discussion

This is the first study to comprehensively analyse the role of p110δ, a catalytic subunit belonging to class I PI3Ks, in granulocytic functions in mice. To that end, we employed a genetically engineered model, using mice devoid of p110δ subunit activity in all tissues. We found that selected antimicrobial functions (NET release, phagocytosis, degranulation and production of ROS) remained unaltered in p110δ^D910A/D910A^ mice as compared to WT littermates. Similarly, the survival of neutrophils in cultures was unchanged in p110δ^D910A/D910A^ mice. Furthermore, the lack of the p110δ subunit only slightly abrogated bacterial killing and decreased the potential to release cytokines, specifically, IL-17, IL-1β and TNF-α, in BM granulocytes. Notably, we observed differences in the activation state of splenic or BM granulocytes and identified distinct subpopulations of granulocytes in the bone marrow and in the spleen of KO mice, distinguished based on the expressions of CD80, CD83 and CD86. 

Our observations indicate that the weights of the spleens of WT mice were significantly higher than those in p110δ^D910A/D910A^ mice (Figure 1a and Figure S1a), which is in line with data previously published by Okkenhaug et al. They reported that the spleens of p110δ^D910A/D910A^ mice revealed, on average, 50% fewer cells than the spleens of WT mice [5]. However, by employing flow cytometry analysis, we did not observe any alterations in the percentage of peripheral white blood cell subpopulations, including neutrophils, in p110δ^D910A/D910A^ mice (Figure 1b and Figure S2b). These findings are in accordance with observations by Bucher et al. [7], who reported unaltered percentages and total number of neutrophils in the bone marrow, peripheral blood, spleen and lung of p110δ^D910A/D910A^ mice in comparison to WT mice. Likewise, the same parameters remained unchanged in p110γ KO mice. Strikingly, only animals lacking the expressions of both p110γ and p110δ subunits were characterized by significantly increased total numbers and percentages of neutrophils in blood, spleen and lungs [7]. On the contrary, there are reports that the deletion of p110γ or p110δ alone is sufficient to raise neutrophil counts in the blood [5,8,9]. Existing discordant data preclude drawing final conclusions on the role of specific p110δ subunits in neutrophil homeostasis. Further studies are warranted to elucidate the mechanisms governing the production and release of neutrophils from bone marrow and their survival in the circulation.

In order to evaluate the role of p110δ in neutrophil functions, firstly, we investigated the ability of p110δ^D910A/D910A^ neutrophils to synthesize ROS. It was previously suggested that apart from p110γ, other subunits, including p110δ, are also involved in ROS generation by human neutrophils [10]. The use of isoform-selective PI3K inhibitors revealed that ROS production in human neutrophils is regulated by the temporal control of p110γ and p110δ [3]. Furthermore, Condliffe et al., reported that ROS production in response to fMLP in bone-marrow-derived murine neutrophils was noticeably lower than in human neutrophils and mainly dependent on PI3Kγ alone [11]. In our studies, however, we did not detect any differences in ROS production between WT and p110δ^D910A/D910A^ murine neutrophils stimulated with H_2_O_2_ or PMA.

One of the strategies used by neutrophils in order to contribute to host defence and tissue repair is the exocytosis of a wide array of granule proteins. Therefore, we further investigated the ability of p110δ^D910A/D910A^ neutrophils to release azurophilic granules upon activation (Figure 2c). It was suggested that PI3Ks are involved in neutrophil degranulation, as the use of a PI3K inhibitor led to the downregulation of granule release by neutrophils obtained from allergic patients [12]. In addition, it was demonstrated that the p110δ subunit plays an essential role in chemotaxis [13]. This directional movement, similar to the degranulation process, requires actin polymerization [14]. Even though these two distinct neutrophil activities share the same events of cytoskeletal remodelling, we were unable to detect any significant difference between p110δ^D910A/D910A^ and WT neutrophils in releasing the content of azurophilic granules [6]. Our data are in line with Hoenderdos et al.’s, who reported that under hypoxia conditions, the degranulation of human neutrophils was inhibited by isoform-selective PI3Kγ inhibitors but not by PI3Kδ inhibitors, suggesting a major role of the p110γ subunit in this process [15]. Similarly, Fensome et al. showed that PI3Kγ is required for granule exocytosis in granulocyte-like HL-60 cells [16].

Phagocytosis, as a multi-step process, has been shown to be differentially regulated by various classes of PI3K. Vieira et al. demonstrated that class I PI3Ks participate in the phagocytosis of large particles by macrophages but are not required for phagosomal maturation. On the other hand, class III PI3K is essential for phagolysosome formation, directing the fusion of phagosomes with late endosomes/lysosomes [17]. Studies by Vieira et al., who investigated the effect of genetic ablation of the α and β isoforms of the p85 regulatory subunit of class I PI3Ks, were further complemented by Tamura et al., who provided evidence on the role of specific p110 catalytic subunits in the process of phagocytosis [18]. These authors demonstrated that the p110α subunit but not p110β, p110δ or p110γ is involved in the process of phagocytosis by murine macrophages. Contrastingly to the aforementioned reports, the simple use of wortmannin, which is a pan-class inhibitor of PI3Ks [19], did not affect the phagocytic capabilities of human granulocytes against *Candida albicans* [20]. Similarly, we did not observe any effect of p110δ^D910A/D910A^ on the phagocytic abilities of murine neutrophils. Altogether, these data suggest that phagocytosis in murine granulocytic cells may be a PI3K-independent process, or it may depend on a subtype of p110 other than p110δ. However, one should bear in mind that the method applied in this study only gives us information about the uptake of the pathogen. To test the influence of p110δ on the particular steps of phagocytosis, further investigations are required.

Among their multiple physiological functions, PI3Ks have been implicated in the regulation of NET formation [21,22,23]. The selective inhibition of the p110γ subunit of class I PI3K led to diminished ROS synthesis and dose-dependent decrease in NET release by human granulocytes stimulated with PMA or *Leishmania* *amazonensis* [24]. On the other hand, the same group reported that the involvement of the p110δ subunit was necessary for *L.* amazonensis-induced NET release, and this process was independent of ROS synthesis. The aforementioned findings only partially overlap with our observations. In our study, neither NET formation nor ROS synthesis was attenuated in p110δ^D910A/D910A^ mice as compared to WT mice. One of the reasons for these differences may be the use of various NET inducers. Currently, NET-formation studies state that various inducers may trigger divergent molecular pathways [25]. In our study, we employed PAF and LPS, whilst DeSouza-Vieira et al. [24] stimulated cells with PMA or L. amazonensis promastigotes. Strikingly, they reported that the inhibition of the p110δ subunit was only involved in a parasite- but not PMA-stimulated process, which further underscores the different pathways triggered by distinct stimuli. Furthermore, differences in PI3K activation patterns between murine and human granulocytes have been previously described [11]. Condliffe et al. reported that the primed respiratory burst of granulocytes resulted in the biphasic activation of class I *PI3K* (γ and δ) in humans, while Schepetkin et al. pointed to the dominating role of the p110*δ* subunit in ROS synthesis by human granulocytes. Contradictorily, the synthesis of ROS by tumour necrosis factor (TNF)-α-primed murine neutrophils stimulated with N-formyl-methionyl-leucyl-phenylalanine (fMLP) remained largely dependent on PI3Kγ alone [11]. Studies by Kimura et al. provided further evidence on the involvement of PI3Kγ in NET release. PI3Kγ KO mice exhibited decreased NET formation in vivo and in vitro, and the pharmacological inhibition of PI3Kγ diminished NET formation by both murine and human granulocytes. These authors also pointed to the therapeutic potential of the PI3Kγ-driven blockade of NET release, since it alleviated a pathology mimicking microscopic polyangiitis and reduced anti-neutrophil cytoplasmic antibodies (ANCAs) titers in diseased mice [26]. Altogether, existing data point to the crucial role of PI3Kγ in activated murine granulocytes, which is in contrast with the shared involvement of p110γ and p110δ subunits in human granulocytes. Our data corroborate the redundancy of the functional p110δ subunit for NET release in mice.

In our study, we employed a bacterial killing assay to compare the abilities of p110δ^D910A/D910A^ and WT neutrophils to kill bacteria. It was previously shown that macrophages from p110δ^D910A/D910A^ mice revealed augmented toll-like receptor signalling and defective bactericidal activity [27]. Our results indicate that a similar effect could be observed in p110δ^D910A/D910A^ neutrophils, as the ability to kill *E. coli* of WT neutrophils tended to be higher than that of KO neutrophils. However, this difference was not statistically significant. 

Under physiological conditions, neutrophils undergo constitutive or spontaneous apoptosis to maintain cellular homeostasis. During inflammation, extending the lifespan of neutrophils is essential for the efficient destruction of pathogens [28]. It is well documented that some bacteria-derived products, such as lipopolysaccharides (LPS), proinflammatory cytokines, chemokines or growth factors, including granulocyte macrophage-colony stimulating factor (GM-CSF), delay neutrophil apoptosis [29]. It was suggested by Juss et al. that PI3K isoforms, especially p110γ and p110δ, may sustain the GM-CSF-stimulated survival of neutrophils [30]. Interestingly, they observed that the pharmacological inhibition of any single subunit of PI3K class I does not abrogate the GM-CSF survival response of human neutrophils [30]. The abrogation of this pro-survival effect of GM-CSF could only be observed when multiple, at least three, PI3K Class I isoforms were inhibited at the same time. Similarly, the genetic ablation of p110γ or both p110γ and p110δ subunits did not affect the magnitude of the GM-CSF-mediated survival response of neutrophils [30]. These reports are entirely consistent with the lack of effects of p110δ functional knockout on neutrophil survival seen in our study.

It has been shown that neutrophils may act as antigen-presenting cells and have the potential to express costimulatory molecules, such as CD80 and CD86, as well as dendritic-cell activation marker CD83 [31,32]. Importantly, PI3K was pointed out to be crucial for the maturation of dendritic cells (DCs). It was shown that the expressions of monocytic (m)DC-specific markers such as CD80 and CD83 was significantly reduced in the presence of LY294002, a PI3K inhibitor [33]. Similarly, a study by Xue et al. revealed that another inhibitor of PI3K, ZSTK474, supressed mDC differentiation, causing a significant dose-dependent reduction in CD80, CD86 and CD83 expressions [34]. Furthermore, Liu et al. reported that the use of a PI3K inhibitor reduced the expression of CD83 and showed that a non-specific PI3K inhibitor blocked the up-regulation of CD86 in mDCs [35]. As PI3K is involved in the activation of neutrophils, we found it interesting to evaluate the expressions of the aforementioned activation markers in BM and splenic neutrophils in p110δ^D910A/D910A^ and WT mice. We observed that splenic residual p110δ^D910A/D910A^ neutrophils expressed CD80 at a lower level than WT cells, which is in line with current studies conducted with the use of human mDCs. We also observed a slightly higher expression of CD80 in BM neutrophils and CD86 in splenic neutrophils in p110δ^D910A/D910A^ mice as compared with WT mice, as well as a diminished expression of CD86 by the BM granulocytes of p110δ^D910A/D910A^ mice (of note, these differences did not reach statistical significance). In our studies, we did not detect differences in CD83 expression neither in BM nor in splenic neutrophils, which is contrary to the aforementioned reports [33,34,35]. This discrepancy may be an effect of the selected models (murine cells vs. human blood/human cord blood cells) as well as the type of cells, since most of the studies concerning the aforementioned markers were conducted with the use of human monocytes differentiated into DCs. Interestingly, we observed a divergence between the activation status of BM and splenic granulocytes. This phenomenon can be associated with the fact that splenic neutrophils express different functionalities from circulating neutrophils [36]. Interestingly, the multiparametric tSNE analysis revealed the presence of distinct subpopulations of neutrophils, unique for p110δ^D910A/D910A^. These data underline that the lack of functional p110δ has an effect on the phenotype of neutrophils, which may result in a not fully effective immune response.

The catalytic p110 subunits of class I PI3K are membrane lipid kinases classically involved in signal transduction. It was reported that a PI3K inhibitor, IC87114, highly selective for p110δ, significantly reduced TNF secretion by murine macrophages [37]. These results provided evidence that PI3K, specifically, its p110δ isoform, modulates the constitutive secretion of TNF. In line with this, Fortin et al. established that the involvement of PI3K in chemokine production in neutrophils can be ascribed to p85α and p110δ subunits [38]. Moreover, studies with azithromycin and monocytes from systemic lupus erythematosus patients revealed that the PI3K signalling pathway is involved in the regulation of cytokine secretion (IL-6, IL-1β, IL-10) [39]. Recent studies on the regulation of antiviral responses highlighted the role of the p110δ subunit in shaping the cytokine profile in infected lungs (including the synthesis of IL-6, IFNβ and IFNλ_1/3_) [40]. Based on these results, we determined the level of cytokines associated with inflammatory response, such as TNF-α, IFN-γ, IL-6, IL-1β, IL-17 and IL-10, in the neutrophils of p110δ^D910A/D910A^ and WT mice. Our results are partially in line with the aforementioned studies, as we detected a lower production of IL-17, IL-1β and TNF-α in the BM neutrophils of p110δ^D910A/D910A^ mice. We did not reveal any differences in the production of IFN-γ, IL-6 and IL-10 by BM neutrophils nor any dissimilarity in cytokine production by splenic neutrophils between WT and p110δ^D910A/D910A^ mice. Similar to these observations, the study by Okeke et. al. did not detect differences in the levels of IL-6 in the serum or peritoneal fluids of p110δ^D910A/D910A^ and WT mice after LPS challenge [41]. However, this group detected a lower level of IL-10 in the serum of WT mice than in that of p110δ^D910A/D910A^ mice, 2 h post LPS challenge. Such discrepancy may be a result of the various experimental settings and methodology used.

Altogether, our data obtained from assays measuring the antimicrobial functions of neutrophils did not reveal significant changes between p110δ^D910A/D910A^ and WT mice. However, we observed differences between the studied groups when the expressions of CD80 and CD86 activation markers as well as the production of cytokines were analysed. Moreover, the tSNE analysis demonstrated the presence of distinct subpopulations of neutrophils, unique for p110δ^D910A/D910A^. It cannot be excluded that the observed differences impacted the presentation of antigens or proinflammatory response by neutrophils in response to pathogens under in vivo conditions.

## 4. Materials and Methods 

### 4.1. Reagents

Foetal bovine serum (P30-5500; FBS) was purchased from Pan-Biotech (Aidenbach, Germany). Platelet activating factor (60900; PAF) was purchased from Cayman Chemicals (Ann Arbor, Michigan, United States), hydrogen peroxide (BA5193111; H_2_O_2_) from POCH (Gliwice, Poland), mouse GM-CSF (415-ML) from R&D Systems (Minneapolis, MN, USA), HEPES (BE17-737E) from Lonza (Basel, Switzerland) and mouse C5a (HC1101) from Hycult Biotech (Uden, The Netherlands). Roswell Park Memorial Institute (11835-063; RPMI) 1640 medium, SYTOX Orange (S11368), ionomycin (I4222) and *E. coli* BioParticles (E2861; K-12 strain) were purchased from Thermo Fisher Scientific (Waltham, Massachussets, United States). Calcium ionophore A23187 (C7522; CI), lipopolysaccharides (L2755; LPS) isolated from *E. coli*, Cytochalasin B (C6762) from *Drechslera dematioidea*, dihydrorhodamine 123 (D1054), DNAse (DN25) and phorbol 12-myristate 13-acetate (P1585, PMA) were purchased from Sigma-Aldrich (St. Louis, MO, USA). All other reagents, if not stated otherwise, were purchased from Becton Dickinson (BD; Franklin Lakes, NJ, USA).

### 4.2. Mice

Mice used in the study expressing a catalytically inactive form of p110δ (p110δ^D910A^) were created by point mutation to prevent changes in the expression levels of the other PI3K catalytic and regulatory subunits [5]. Expression of the mutated p110δ protein was equivalent to the wild-type protein, as were the expressions of the other PI3K subunits [5]. P110δ ^D910A/D910A^ lipid kinase activity was abolished in p110δ ^D910A/D910A^ mice, with no changes in the kinase activities of p110α and p110β [5]. Mutant mice and their littermates backcrossed to the wild type (WT) Balb/c genetic background were kindly provided by prof. Bart Vanhaesebroeck of UCL Cancer Institute London, UK. Mice were kept and bred in individually ventilated cages in the institutional animal facility; litters were genotyped by polymerase chain reaction. Animal housing and handling were performed in accordance with institutional and governmental ethical and legal regulations governing animal care. Animals were used for experiments at the age of 8–12 weeks.

### 4.3. Blood Collection and Staining

Blood samples were collected by cardiac puncture into EDTA-coated probes. Then, 50 µL of blood sample was incubated with 25 µL of blocking buffer (20% rat serum and 20% FBS in PBS) for 10 min at room temperature (RT). After incubation, surface antigens were stained with the following antibodies: anti-CD11b BV510 (562950; BD Horizon; 1:400), anti-CD45 V500 (562129; BD Horizon; 1:200), anti-Ly6G BV605 (563005; BD Horizon; 1:200) and Ly6C BV711 (128037; BioLegend (San Diego, CA, USA); 1:400) for 30 min at room temperature (RT) in the dark. Subsequently, 500 µL of RBC Easy Lyse 1× solution (S2364; Dako (Glostrup, Denmark)) was added to the samples to lyse erythrocytes, and the samples were lysed for 15 min at 4 °C. After passing the cells through a 100 μm strainer, the percentages of peripheral blood leukocytes subpopulations were analysed with a BD LSRFortessa flow cytometer.

### 4.4. Cell Isolation

Bone marrow cells were harvested from murine femurs, tibias, hips and humeri. Animals were euthanized, the skin and the muscles were removed from both legs and arms using scissors. Subsequently, the acetabula were dislocated from the hip joints, and feet were cut at the ankle joints. Afterward, most of the flesh from the bones was removed, and bones were collected in falcon tubes filled with 2% FBS in PBS that were then placed on ice until further processing. Bones were then placed in a sterile petri dish containing 2% FBS in PBS. Epiphyses of the bones were cut off; then, the bone marrow cells were collected by flushing the bones with 5–10 mL of 2% FBS in PBS using a 26-gauge needle and then filtered through a sterile 100 μm cell strainer into the falcon tube. Subsequently, bone marrow cell suspensions were washed, and neutrophils were negatively isolated from bone marrow suspensions using an EasySep Mouse Neutrophil Enrichment Kit (19762; Stemcell Technologies(Vancouver, British Columbia, Canada)).

Harvested murine spleens were weighed and placed into the 100 μm cell strainer. Each spleen was gently mashed using the plunger end of the syringe into the falcon tube placed on ice. Splenic cells were centrifuged at 500× *g* for 5 min and suspended in 5 mL of 1× Lysis Buffer (Pharm Lyse BD) to remove red blood cells. After 5 min of incubation at RT in the darkness, cells were centrifuged at 200× *g* for 5 min and then washed in 5 mL of 1% FBS in PBS. Subsequently, murine spleen cells were suspended in PBS (>1 × 10^5^/mL), and viable cells were counted using trypan blue staining solution.

### 4.5. Analysis of Neutrophil Activation Markers

Splenocytes or isolated bone marrow neutrophils were seeded into 96-well plates (1–2 × 10^6^ per well). Cells were centrifuged for 5 min at 350 RCF; then, they were stained with Fixable Viability Stain 440UV (565388; BD Horizon) according to the manufacture’s recommendation and incubated for 6 min at 37 °C, 5% CO_2_. After two washes, the samples were blocked with 5% FBS in PBS for 10 min at RT. Subsequently, surface antigens were labelled using the following antibodies: anti-CD11b BV510 (562950; BD Horizon; 1:400), anti-CD45 V500 (562129; BD Horizon; 1:200), anti-Ly6G BV605 (563005; BD Horizon; 1:200), anti-Ly6C BV711 (128037; BioLegend; 1:400), anti-CD80 Pe-Cy7 (104734; BioLegend; 1:200), anti-CD83 APC (558206; BD Pharmingen; 1:200), anti-I-A/I-E BB700 (746197; BD OptiBuild; 1:200) and anti-CD86 FITC (561962; BD Pharmingen; 1:200) for 30 min at RT. Cells were then fixed using BD Cell fix and analysed using a BD LSRFortessa flow cytometer.

### 4.6. Degranulation Assay

A degranulation assay was carried out as previously described in [42].

### 4.7. Phagocytosis Assay

Neutrophils (3 × 10^5^ cells) were incubated with 25 µg of *E. coli* bioparticles for 30 min at 37 °C, 5% CO_2_. Subsequently, trypan blue at the final concentration of 0.01% was added to quench the fluorescence of non-phagocyted bioparticles; cells were washed, and the percentage of cells phagocyting *E. coli* bioparticles was analysed with a BD LSRFortessa flow cytometer.

### 4.8. Neutrophil Extracellular Trap Formation Assays

Isolated neutrophils were suspended in RPMI-1640 medium without phenol red with 10 mM HEPES and seeded into the wells of 24-well plates (5 × 10^4^ cells per well) for the measurement of extracellular DNA release or into 8-well Lab-Tek chambers (2.5 × 10^4^ per chamber) for NET immunostaining. Cells were allowed to settle for 30 min at 37 °C, 5% CO_2_ and then stimulated with 2 µM calcium ionophore A23187 (CI), 5 µM platelet activating factor (PAF) or 20 µg/mL lipopolysaccharides (LPS) isolated from *E. coli*. After 3 h of stimulation, NET formation was assessed quantitatively (DNA release measurement) and qualitatively (NET immunostaining) as described previously [21], with minor modifications. To measure extracellular DNA release, 10 U/mL DNAse was added to the wells for 10 min, and samples were further processed as described in [21]. Lab-Tek samples were prepared for immunolabeling, and NET components were stained with anti-neutrophil elastase antibodies (Abcam (Cambridge, United Kingdom); ab21595; 1:100) and secondary antibodies conjugated with FITC (Goat Anti-Rabbit IgG H&L; Abcam; ab6717; 1:1000) and SYTOX Orange DNA-binding dye [21].

### 4.9. Reactive Oxygen Species Formation Assay

To analyse the respiratory burst in murine neutrophils, 1 × 10^5^/mL cells per well were seeded into black 96-well plates. Cells were incubated with 4 μg/mL dihydrorhodamine 123 (DHR 12; Thermo Fisher Scientific, Waltham, MA, USA) in the dark for 30 min at 37 °C, 5% CO_2_. After incubation, excess reagent was washed out, and cells were stimulated with PMA (100 nM) and H_2_O_2_ (100 μM). Fluorescence was monitored every 15 min for 4 h in the FLUOstar OMEGA plate reader.

### 4.10. Analysis of Cell Survival

The analysis of cell survival was performed as previously described [6] with minor modifications. Isolated granulocytes were resuspended in RPMI 1640 supplemented with 10% FBS and antibiotics–antimycotics and seeded into 48-well plates (5 × 10^5^ cells per well). Subsequently, the cells were stimulated with LPS isolated from *E. coli* (100 ng/mL), GM-CSF (10 ng/mL) or C5a (10 ng/mL) and incubated for 24 h at 37 °C, 5% CO2. After incubation, the cells in the wells were vigorously pipetted and collected into tubes, which were centrifuged at 300× *g* for 5 min. Granulocytes were resuspended in 30 µL of blocking buffer (5% rat serum and 5% FBS in PBS) and incubated for 10 min at RT. Then, surface-cell antigens were stained with anti-CD45 V500 (562129; BD Horizon; 1:200) and anti-Ly6G BV605 (563005; BD Horizon; 1:200) antibodies and incubated for 30 min at RT in the dark. Next, samples were diluted with 1% FBS in PBS, and propidium iodide (BMS500FI/300CE; eBioScience (San Diego, CA, USA)) was added to the samples at the final concentration of 2 µg/mL. Cells were immediately passed through cell strainers, and their viability was analysed with a BD LSRFortessa flow cytometer.

### 4.11. Ex Vivo Stimulation of Cells and Intracellular Cytokine Staining

Splenocytes or isolated granulocytes were resuspended in RPMI 1640 supplemented with 10% foetal bovine serum (FBS) and antibiotics–antimycotics and seeded into the wells of 96-well plates (2–3 × 10^6^ cells per well). Cells were stimulated with 20 ng/mL phorbol 12-myristate 13-acetate (PMA) + 1 µg/mL ionomycin for 1 h at 37 °C, 5% CO_2_; then, BD GolgiStop protein transport inhibitor was added to the wells according to the manufacturer’s instructions, and cells were incubated for 4 h at 37 °C, 5% CO_2.._ After incubation, plates were stored overnight at 4 °C. The following day, samples were centrifuged and stained with Fixable Viability Stain 440 UV (565388; BD Horizon) according to the manufacturer’s recommendations. Subsequently, the samples were blocked with 5% FBS in PBS for 10 min at RT; then, surface antigens were stained with the following antibodies: anti-CD11b BV510 (562950; BD Horizon; 1:400), anti-CD45 V500 (562129; BD Horizon; 1:200), anti-Ly6G BV605 (563005; BD Horizon; 1:200) and anti-Ly6C BV711 (128037; BioLegend; 1:400) for 30 min at RT. The cells were then fixed and permeabilized with Cytofix/Cytoperm (554722; BD Bioscience). Intracellular cytokines were stained with the following cocktail of antibodies: anti-TNF AF700 (558000; BD Pharmingen; 1:200), anti-IFN-γ APC (554413; BD Pharmingen; 1:200), anti-IL-6 FITC (11-7061-41; eBioscience; 1:400), anti-IL-1β PE (12-7114-80; eBioscience; 1:800), anti-IL-10 PerCP-Cy5.5 (505028; BioLegend; 1:400) and anti-IL-17A APC-Cy7 (560821; BD Pharmigen; 1:200) for 30 min at 4 °C. Cells were resuspended in 1% FBS in PBS and analysed with a BD LSRFortessa flow cytometer using Diva software.

### 4.12. Bacterial Killing Assay

A single colony of *Escherichia coli* (ATCC^®^ 25922™) was cultured overnight in Luria Broth medium at 37 °C with gentle shaking. The overnight bacterial culture was diluted to 1:100 in LB medium, grown for the subsequent 2–3 h under the same conditions, centrifuged and resuspended in physiological saline at the density of 0.5 McFarland. 

Murine neutrophils were seeded into 48-well plates (2 × 10^4^ cells per well) in RPMI-1640 medium without phenol red with 10 mM HEPES and allowed to settle for 30 min. Subsequently, heat-inactivated mouse serum (at the final concentration of 2%) and *E. coli* bacteria were added to the neutrophils at the multiplicity of infection 10:1 (*E. coli*: neutrophils). Plates were centrifuged at 300× *g* for 5 min and incubated for 1 h at 37 °C, 5% CO_2_. Extracellular bacterial killing was assessed after addition of 100 U/mL DNase to the wells for 10 min (37 °C). Supernatants were collected and serially diluted in physiological saline. Aliquots (50 μL) of bacterial suspension were seeded in triplicates on LB agar and grown overnight, and colonies were counted. The number of colonies grown from samples containing neutrophils and bacteria was divided by the number of colonies grown from samples where only bacteria were present, and the results are presented as percentages.

### 4.13. Statistical Analysis

All statistical analyses were performed using GraphPad Prism Software v. 6 (GraphPad Software, La Jolla, CA, USA). Unless otherwise stated, data are expressed as means + SEM. Routinely, the normality of the data distribution was tested using a Kolmogorov–Smirnov test. Unless stated otherwise, multiple groups were compared with the use of the ANOVA test or its modifications with appropriate post hoc tests, whilst two groups were compared with the use of *t*-tests. *p*-values of ≤0.05 were considered statistically significant.

## 5. Conclusions

Several studies have reported that the inactivation of the catalytic subunit of p110δ resulted in impaired immune responses. Therefore, we performed a comprehensive analysis of neutrophil functions such as phagocytosis, degranulation, ROS production, NET formation and ability to kill bacteria, as well as an analysis of the residual neutrophil activation status and cytokine production in p110δ mutant mice. We did not observe any alterations in any of the following antimicrobial functions of p110δ^D910A/D910A^ mice: ROS synthesis, degranulation, NET release and phagocytic activity. The functional knock-out of p110δ did not affect the survival of isolated granulocytes in vitro. However, we detected a significantly higher percentage of CD80-positive cells in the spleen and a slightly higher percentage of CD86-positive granulocytes in the bone marrow of WT than in p110δ^D910A/D910A^ mice. We also observed a slightly higher expression of CD80 in the BM neutrophils and CD86 in the splenic neutrophils of p110δ^D910A/D910A^ mice than in those of WT mice. Additionally, we noticed that the ability to kill *E. coli* of WT neutrophils tended to be higher than that of p110δ^D910A/D910A^ neutrophils, similar to the production of cytokines (IL-17, IL-1β and TNF-α). Altogether, our data indicate that the p110δ subunit is not a key player in most neutrophil functions. However, the observed differences between the studied groups suggest that neutrophils lacking an active p110δ subunit may respond differently to pathogens in vivo. In the light of our studies and other literature reports, the p110γ subunit seems to be a major PI3K subunit regulating granulocytic functions in mice. Our observation is of great importance in terms of therapeutic approaches, since PI3K isoforms represent attractive therapeutic targets in inflammation, and several inhibitors have already entered phase I clinical trials. Our attention should be focused on describing the differences in the role of p110δ in granulocytic functions between humans and mice. Accordingly, murine models do not seem to be the best choice in order to perform studies on potential therapies targeting p110δ intended for humans.

## Data Availability

The data presented in this study are available on request from the corresponding author.

## Supplementary Materials

Supplementary materials can be found at https://www.mdpi.com/article/10.3390/ijms23126361/s1.

## Abbreviations

ANCAs ATCC	Anti-neutrophil cytoplasmic antibodies American Type Culture Collection
BM	Bone marrow
DCs	Dendritic cells
FBS	Foetal bovine serum
GM-CSF	Granulocyte-macrophage colony-stimulating factor
IFN	Interferon
IL	Interleukin
LB	Luria broth
LPS	Lipopolysaccharide
MFI	Median fluorescence intensity
NETs	Neutrophil extracellular traps
PAF	Platelet activating factor
PI3K	Phosphoinositide 3-kinases
PMA	Phorbol 12-myristate 13-acetate
ROS	Reactive oxygen species
RPMI	Roswell Park Memorial Institute
TNF	Tumour necrosis factor
WT	Wild type
