# Supplementary material for: Lack of Functional P110δ Affects Expression of Activation Marker CD80 but Does Not Influence Functions of Neutrophils
Source: Int J Mol Sci. 2022 Jun 7;23(12):6361. doi: 10.3390/ijms23126361 (PMC9223848; doi:10.3390/ijms23126361)
Supplement: Supplementary file 1 [file ijms-23-06361-s001.zip › ijms-1721655-supplementary.pdf]

Supplementary Materials

Lack of functional p110 δ affects expression of activation marker CD80 but does not influence functions of neutrophils

Aneta Manda-Handzlik [1], Agnieszka Mroczek [1, 2], Weronika Kuzmicka [1], Adrianna Cieloch [1, 2], Zuzanna Homoncik [1, 3], Angelika Muchowicz [4, 5], Urszula Demkow [1], Małgorzata Wachowska[1]*

[1] Department of Laboratory Diagnostics and Clinical Immunology of Developmental Age, Medical University of Warsaw, Zwirki i Wigury 63a Street, 02-091 Warsaw, Poland; aneta.manda-handzlik@wum.edu.pl (A.M-H.); agnieszka.mroczek@wum.edu.pl (A.M.M.); weronika.kuzmicka@wum.edu.pl (W.K.); adrianna.cieloch@wum.edu.pl (A.C.); homoncik.zuzanna@gmail.com (Z.H.), urszula.demkow@uckwum.pl (U.D.);

[2] Doctoral School, Medical University of Warsaw, Zwirki i Wigury 61 Street, 02-091 Warsaw, Poland;

[3] Student's Scientific Group at Department of Laboratory Diagnostics and Clinical Immunology of Developmental Age, Medical University of Warsaw, 02-091 Warsaw, Poland.

[4] Department of Immunology, Medical University of Warsaw, Jana Nielubowicza 5 Street, 02-097, Warsaw, Poland; angelika.muchowicz@wum.edu.pl (A.M.);

[5] Department of Clinical Immunology, Medical University of Warsaw, Nowogrodzka 59 Street, 02-006, Warsaw, Poland

* Correspondence: malgorzata.wachowska@wum.edu.pl;

a

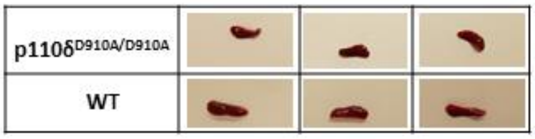

b

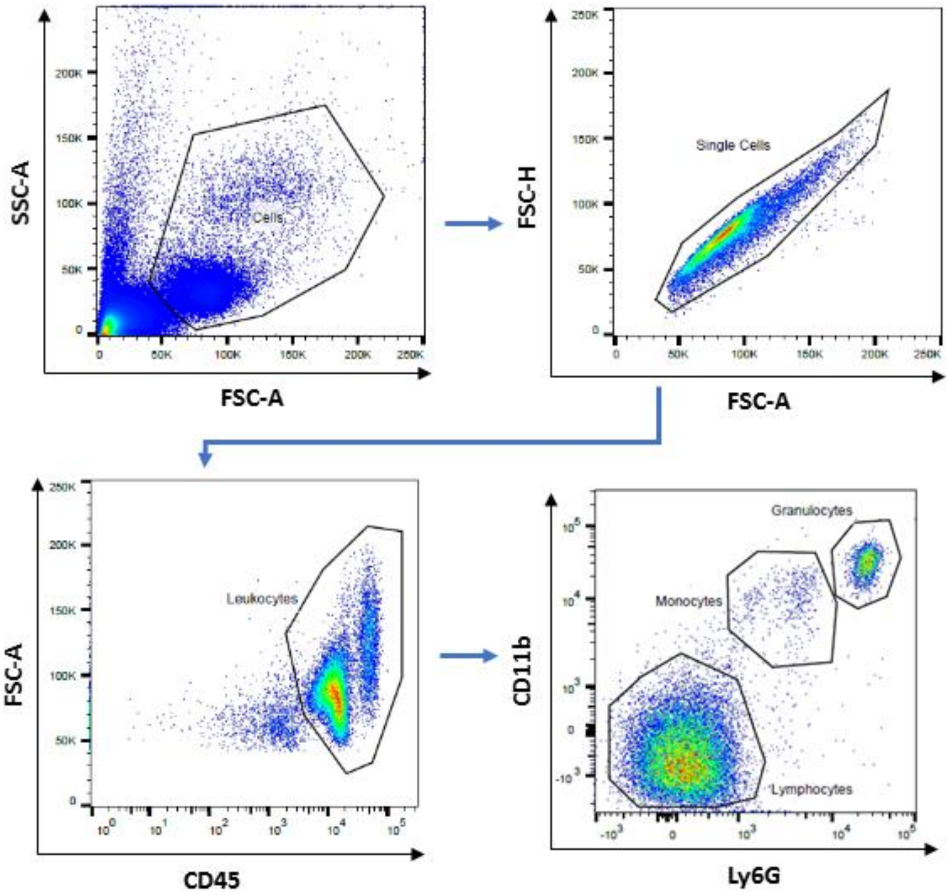

Supplementary Figure S1 (a) Representative photos of spleens from wild-type (WT) and p110δ$^{D910A/D910A}$. **(b)** Gating strategy used for the analysis of different types of white blood cells isolated from p110δ$^{D910A/D910A}$ mice. Arrows show the sequence of the gating used, starting from single cells gate. Cells were plotted as FSC versus CD45 to identify leukocytes and CD11b versus Ly6G to identify lymphocytes, monocytes and granulocytes.

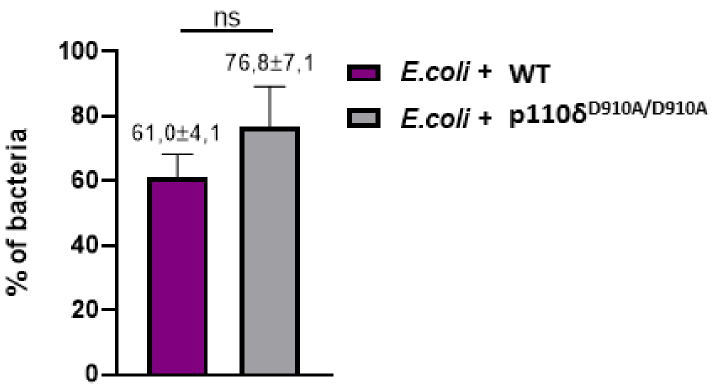

Supplementary Figure S2 Extracellular bactericidal activity of neutrophils isolated from WT and p110δ$^{D910A/D910A}$ mice against *E.coli*. Granulocytes were incubated with bacteria for 1 hour, supernatants were collected, serially diluted, cultured and colony forming units (CFU) were counted the next day. CFU counts in each sample were divided by the number of colonies grown from the samples, where no granulocytes were added, and were shown as percentage. Results are presented as means + SEM and were analyzed by *t*-test, n=3.

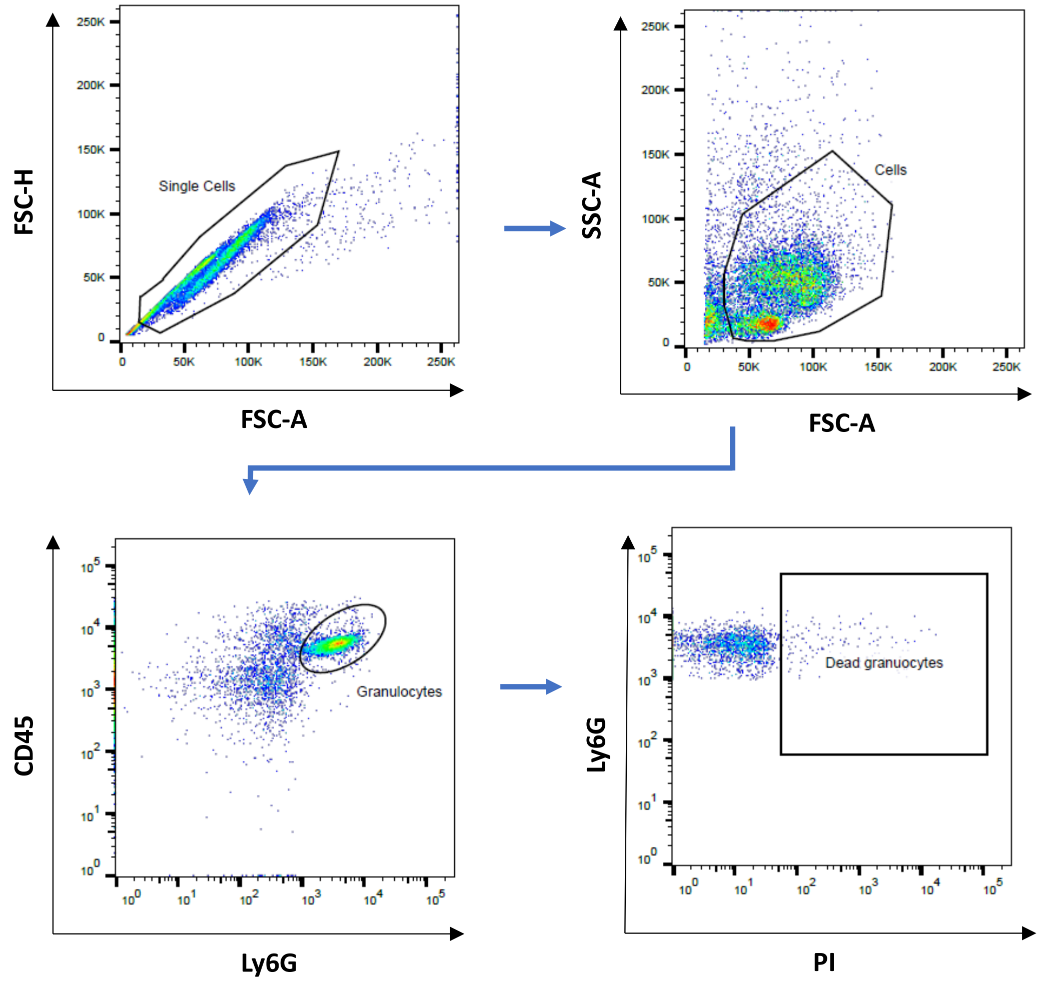

Supplementary Figure S3 Gating strategy used for the survival analysis of neutrophils isolated from p110δ$^{D910A/D910A}$ mice. Arrows show the sequence of the gating used, starting from single cells gate. Cells were plotted as CD45 versus Ly6G to identify neutrophils and Ly6G versus PI to identify death granulocytes.

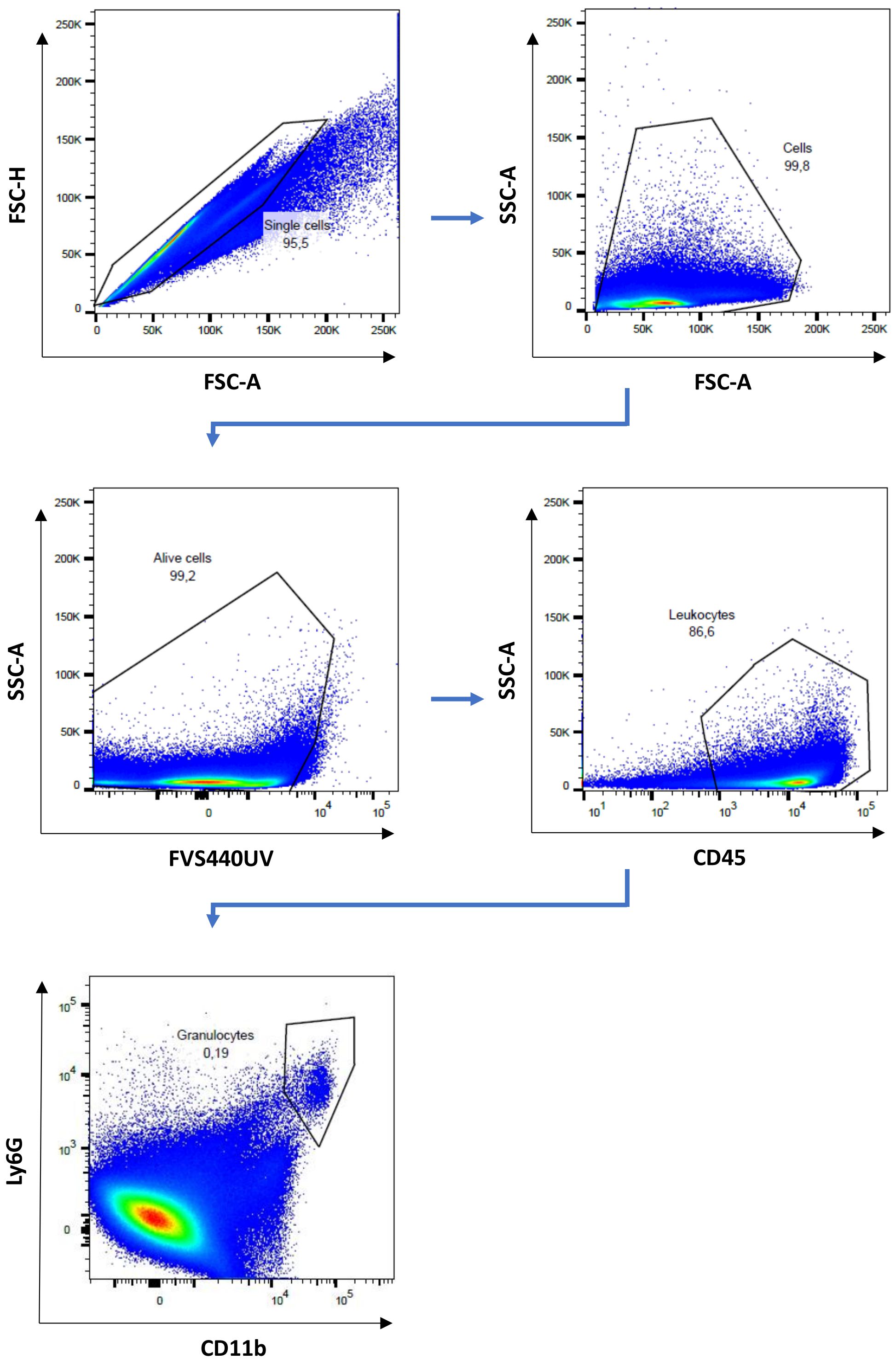

Supplementary Figure S4 Gating strategy used for the analysis of neutrophils isolated from p110δ$^{D910A/D910A}$ mice spleen. Arrows show the sequence of the gating used, starting from single cells gate. Cells were plotted as SSC versus FVS440UV to identify alive cells, SSC versus CD45 to identify leukocytes and Ly6G versus CD11b to identify granulocytes.
